# Standardization of in-house anti-IgG and IgA ELISAs for the detection of COVID-19

**DOI:** 10.1371/journal.pone.0287107

**Published:** 2023-06-09

**Authors:** Kamonthip Rungrojcharoenkit, Rungarun Suthangkornkul, Darunee Utennam, Darunee Buddhari, Soontorn Pinpaiboon, Duangrat Mongkolsirichaikul, Stefan Fernandez, Anthony R. Jones, Thomas S. Cotrone, Taweewun Hunsawong

**Affiliations:** 1 Department of Virology, Armed Forces Research Institute of Medical Sciences (AFRIMS), Bangkok, Thailand; 2 Research Division, Royal Thai Army-Armed Forces Research Institute of Medical Sciences (RTA-AFRIMS), Bangkok, Thailand; 3 Department of Internal Medicine, Kamphaeng Phet Provincial Hospital (KPPH), Kamphaeng Phet, Thailand; University of Bologna / Romagna Local Health Authority, ITALY

## Abstract

Severe acute respiratory syndrome coronavirus 2 (SARS-CoV-2) is the causative agent of coronavirus disease 2019 (COVID-19). RT-PCR detection of viral RNA represents the gold standard method for diagnosis of COVID-19. However, multiple diagnostic tests are needed for acute disease diagnosis and assessing immunity during the COVID-19 outbreak. Here, we developed in-house anti-RBD IgG and IgA enzyme-linked immunosorbent assays (ELISAs) using a well-defined serum sample panel for screening and identification of human SARS-CoV-2 infection. We found that our in-house anti-SARS-CoV-2 IgG ELISA displayed a 93.5% sensitivity and 98.8% specificity whereas our in-house anti-SARS-CoV-2 IgA ELISA provided assay sensitivity and specificity at 89.5% and 99.4%, respectively. The agreement kappa values of our in-house anti-SARS-CoV-2 IgG and IgA ELISA assays were deemed to be excellent and fair, respectively, when compared to RT-PCR and excellent for both assays when compared to Euroimmun anti-SARS-CoV-2 IgG and IgA ELISAs. These data indicate that our in-house anti-SARS-CoV-2 IgG and IgA ELISAs are compatible performing assays for the detection of SARS-CoV-2 infection.

## Introduction

Severe acute respiratory syndrome coronaviruses 2 (SARS-CoV-2) is the causative agent of coronavirus disease 2019 (COVID-19). Since the start of the outbreak in China on December 2019, SARS-CoV-2 has spread rapidly across the globe, causing significant morbidity and mortality, requiring mass vaccination campaigns that today have reached over 60% of the global population [1,2]. SARS-CoV-2 is a single-stranded RNA virus belonging to the β-coronavirus genus of coronaviruses. Its genome encodes non-structural proteins that facilitate the viral replication cycle as well as four structural proteins. These structural proteins include nucleocapsid protein (N), envelope (E), membrane (M) and spike (S). The S protein is of particular relevance, as it is the protein that binds to host cell receptors and facilitates viral entry into host cells. More specifically, the S protein is composed of two subunits, S1 and S2, each playing important roles during the virus replication cycle. The S1 subunit contains a receptor-binding domain that binds to human receptor angiotensin converting enzyme 2 (ACE2). Once bound to ACE2, the S2 subunit mediates the fusion of the virus to the host cell membrane [3]. The S protein (especially the receptor binding domain (RBD) of the S1 subunit) is widely used as the target for vaccine development [4] as well as in assays for antigen or antibody detection [5,6].

Viral genome detection by RT-PCR is the gold standard method for COVID-19 diagnosis. However, the narrow period of viremia limits the practical applicability of this assay. Serological antibody testing can be used as a complementary assay to fill this gap even after symptoms disappear. Beyond the use as a supplementary diagnostic tool, serological tests can also provide useful epidemiological data such as background seroprevalence during outbreaks or determinations of vaccine-derived immunity. In this study, we standardized enzyme-linked immunosorbent assays (ELISAs) using SARS-CoV-2 spike RBD as antigen to detect anti-SARS-CoV-2 IgG and IgA antibodies. Standardization of these assays was based on a well-defined serum sample panel. This panel included SARS-CoV-2 confirmed positive samples (collected during SARS-CoV-2 outbreak) and negative samples collected before and after the 2019 SARS-CoV-2 outbreak.

## Materials and methods

### Serum samples

A panel of SARS-CoV-2 negative and positive serum samples was used in this study to standardize the ELISAs. This panel (Table 1) was composed of human serum samples obtained from Thai patients under a research study approved by AFRIMS Institutional Review Board (IRB), Walter Reed Army Institute of Research (WRAIR No.2836) and two local IRBs including the Royal Thai Army (RTA, RF02_2563) and Institute for the Development of Human Research Protection, Ministry of Public Health, Thailand (IHRP No. 119–2563). The PCR- and/or ELISA-confirmed SARS-CoV-2 negative and positive coded human sera included in this study were collected between 7 and 14 days post onset of symptoms (POS) with written informed consent allowing for future research use. No serum from SARS-CoV-2 vaccinated individuals was included in this study.

**Table 1:** Serum panel.

Group	Euroimmun IgG-confirmed positive(n) (Serum)	Euroimmun IgA-confirmed positive(n) (Serum)	RT-PCR-confirmed positive(n) (Nasal swab)
Negative group (Total, n = 170)			
1. Pre-outbreak serum samples	0/74	0/74	N/A
2. Post-outbreak serum samples	0/96	0/96	0/96
Positive group (Total, n = 46)			
1. RT-PCR positive serum samples	18/18	7/18 (3*)	18/18
2. RT-PCR negative serum samples	28/28	12/28 (1*)	0/28

(*Borderline results were not used in final analysis).

Negative group (IgG ELISA and/or SARS-CoV-2 RT-PCR negative samples) were collected either prior to the SARS-CoV-2 outbreak (n = 74, collected from febrile illness patients who lived in Kamphaeng Phet province during 2016–2019), or after (n = 96, collected from a SARS-CoV-2 surveillance study in September to October of 2020). All negative samples (n = 170) were negative by Euroimmun IgG and IgA ELISAs.

The positive group samples (n = 46) were collected between September and October 2020 from subjects that had symptoms of respiratory illness and were suspected of SARS-CoV-2 infection. Of these, 18 were confirmed positive by RT-PCR assay in respiratory samples (nasal swab), and 28 were RT-PCR negative. All positive samples (n = 46) were positive by Euroimmun IgG ELISA and 19 were Euroimmun IgA ELISA confirmed positive (n = 7; RT-PCR/IgA ELISA confirmed positive and n = 12; IgA ELISA confirmed positive only).

### Real-time reverse transcription polymerase chain reaction (rRT-PCR)

Respiratory specimens were tested for SARS-CoV-2 viruses by rRT-PCR, as described in the US Centers for Disease Control and Prevention (CDC) protocol [7]. Briefly, all swab samples were heat-inactivated at 60°C for 30 minutes before use. Viral RNA was extracted using commercially available RNA extraction kits (Qiagen, Hilden, Germany) according to the manufacturer’s instruction. The extracted viral RNA of each samples was amplified by ABI 7500 (Applied Biosystems, CA, USA).

### In-house anti-SARS-CoV-2 IgG and IgA ELISAs

Direct ELISA was developed and standardized to detect the presence of anti-SARS-CoV-2 IgG and IgA antibodies in the sera. Briefly, Ninety-six well plates were coated overnight with 50 μl/well of spike RBD recombinant proteins (Abcam, UK) at the concentration of 0.1 μg per well. The unbounded spike RBD was removed by washing with phosphate buffered saline containing 0.1% Tween20 (PBST) 350 μl/well for 6 times. Non-specific binding was blocked by adding blocking buffer (5% skim milk in PBST) to each well and incubating at room temperature (RT) for 1 hr followed by washing (6X) with PBST. Serum samples were heat-inactivated at 60°C for 30 minutes and diluted with blocking buffer to a 1:100 ratio. Diluted serum was then added to wells in duplicates (100 μl/well). The blocking buffer was used as blank wells. Plates were washed after incubation at RT for 2 hrs. Then, one of two secondary antibodies (either anti-human IgG (γ) antibody peroxidase (1:40000 dilution, Cat. No. 5220–0330, KPL, USA) or anti-human IgA (α) antibody peroxidase (1:10000 dilution, Cat. No. 5220–0360, KPL, USA)) was added to each well (100 μl/well) and incubated at RT for 1 hr. The peroxidase reaction was visualized by adding Sureblue TMB solution (KPL, USA) and incubating in the dark at RT for 15 and 20 min for IgG and IgA ELISAs, respectively. The reaction was stopped by adding 50 μl/well of 0.5 M sulfuric acid. Absorbance at 450 nm was determined with a spectrophotometer. The OD_450_ of blanks were subtracted from OD_450_ of each sample before data analysis.

### Euroimmun IgG and IgA ELISA kits

The Euroimmun SARS-CoV-2 IgG and IgA test kits using S1 as antigen (Cat No. EI 2606-9601G and Cat No. EI 2606-9601A, Lubeck, Germany) were performed following the manufacturer’s instructions to detect specific IgG and IgA antibodies. The ratios of control and sample ODs over calibrator OD were calculated. Results were interpreted by following Euroimmun recommendations: ratios < 0.8 were defined as negative, ratios ≥ 0.8 to < 1.1 were defined as borderline and ratios ≥ 1.1 were defined as positive results.

### Statistical analysis

GraphPad Prism version 9.0 was used to identify and calculate the receiver operating characteristic (ROC) curve, kappa values and 95% confidence intervals (CIs). Kappa values of >0.75, 0.40 to 0.75, and <0.40 were reported for the assay performance as excellent, fair, and poor agreement, respectively [8].

### Ethics statement

The study protocol was reviewed and approved by AFRIMS Institutional Review Board (IRB), Walter Reed Army Institute of Research (WRAIR No.2836) and two local IRBs including the Royal Thai Army (RTA, RF02_2563) and Institute for the Development of Human Research Protection, Ministry of Public Health, Thailand (IHRP No. 119–2563). It was determined to be non-human subject research study as only de-identified samples were utilized.

## Results

### Receiver operating characteristic (ROC), OD distribution, and the sensitivities and specificities of the in-house anti-SARS-CoV-2 IgG and IgA ELISAs

**Fig 1:**
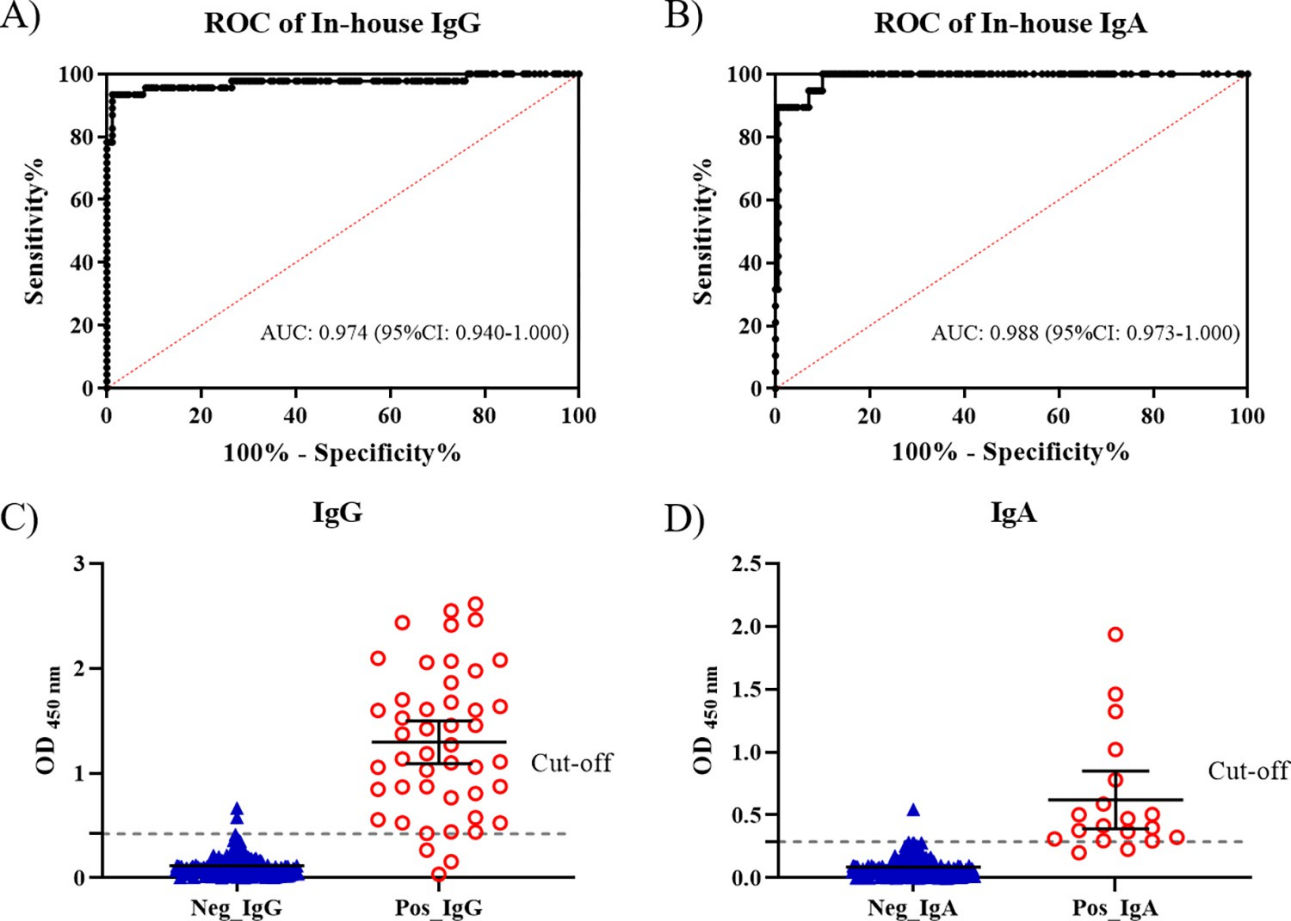
ROC curves and OD distribution. ROC curve analysis using in-house anti-SARS-CoV-2 IgG (Panel A) and IgA (Panel B) ELISAs including the area under the curve (AUC) and 95% confidence intervals. OD distribution of negative control sera and positive SARS-CoV-2 case sera were determined using the in-house anti-SARS-CoV-2 IgG (Panel C) and IgA (Panel D) ELISAs. Black dash line represents positive cut-off.

We created receiver operating characteristic (ROC) curves to identify the optimal positive cut-offs for SARS-CoV-2-infected samples. For ROC curve analysis, 46 and 19 samples that were positive by Euroimmun IgG and IgA ELISAs, respectively, were used to compose a “positive group” whereas 170 samples that tested negative by both RT-PCR and Euroimmun IgG and IgA ELISAs were used to compose a “negative group”. ROC curve and OD distribution of the in-house anti-SARS-CoV-2 IgG and IgA ELISAs were shown in Fig 1. ROC curve analysis indicated that overall assay performance of in-house anti-SARS-CoV-2 IgG (Fig 1A) and IgA (Fig 1B) ELISAs had excellent diagnostic accuracy with an area under the curve (AUC) value of 0.974 (95% confidence interval (95%CI): 0.940–1.000) and 0.988 (95%CI: 0.973–1.000), respectively. The OD distribution of validated samples was shown in a scatter plot with cut-off lines (Fig 1C and 1D for IgG and IgA, respectively).

For the in-house anti-SARS-CoV-2 IgG ELISA (Table 2), we found that OD values 3.6-fold above the mean OD negative control could be used as a positive threshold. The results showed that 43 out of the 46 IgG positive samples were also identified as positive by the in-house assay. Furthermore, only 2 out of the 170 negative samples were identified as positive by the in-house assay. These results of the in-house assay correspond with a sensitivity and a specificity of up to 93.5% and 98.8%, respectively.

**Table 2:** Assay performance for in-house IgG ELISA in comparison to RT-PCR and Euroimmun as standard methods.

Standard method	ELISA	Result	Validated sample		%Sensitivity	%Specificity	Kappa assessment values
			Positive	Negative	(95% CI)	(95% CI)	(95% CI)
RT-PCR	In-house IgG	Positive	18	2	18/18	168/170	0.94
		Negative	0	168	100.0%	98.8%	(0.86–1.00)
					(81.5–100.0)	(95.8–99.9)	excellent
Euroimmun	In-house IgG	Positive	43	2	43/46	168/170	0.93
		Negative	3	168	93.5%	98.8%	(0.87–0.99)
					(82.1–98.6)	(95.8–99.9)	excellent

For the in-house anti-SARS-CoV-2 IgA ELISA (Table 3), OD values 3.2-fold above the mean OD negative control was used as a positive threshold. Among the 19 positive samples, 17 were identified as positive by the in-house assay. Among the 170 negative samples, only 1 were identified as positive by the in-house assay. These results of the in-house assay correspond with a sensitivity and a specificity of up 89.5% and 99.4%, respectively.

**Table 3:** Assay performance for in-house IgA ELISA in comparison to RT-PCR and Euroimmun as standard methods.

Standard method	ELISA	Result	Validated sample		%Sensitivity	%Specificity	Kappa assessment values
			Positive	Negative	(95% CI)	(95% CI)	(95% CI)
RT-PCR	In-house IgA	Positive	7	1	7/18	169/170	0.51
		Negative	11	169	38.9%	99.4%	(0.24–0.78)
					(17.3–64.3)	(96.8–100.0)	fair
Euroimmun	In-house IgA	Positive	17	1	17/19	169/170	0.91
		Negative	2	169	89.5%	99.4%	(0.81–1.00)
					(66.9–98.7)	(96.8–100.0)	excellent

### Assay performance of the in-house anti-SARS-CoV-2 IgG and IgA ELISAs in comparison to RT-PCR and Euroimmun as standard method

The assay performance of our in-house ELISAs to detect anti-SARS-CoV-2 IgG and IgA antibodies was compared with RT-PCR and commercial ELISA kits (Euroimmun anti-SARS-CoV-2 IgG and IgA ELISA kits) (Tables 2 and 3).

Based on the RT-PCR-confirmed COVID-19 positive cases, the sensitivities of in-house anti-SARS-CoV-2 IgG and IgA ELISAs were 100% (18/18) and 38.9% (7/18), respectively. For the assay specificities, the RT-PCR negative SARS-CoV-2 samples were found to be 98.8% (168/170) and 99.4% (169/170) negative when tested by the in-house anti-SARS-CoV-2 IgG and IgA ELISAs, respectively. The agreement kappa values for in-house anti-SARS-CoV-2 IgG and IgA ELISAs were 0.94 (excellent) and 0.51 (fair), respectively.

Using Euroimmun anti-SARS-CoV-2 IgG and IgA ELISAs as standard methods, the sensitivities of in-house anti-SARS-CoV-2 IgG and IgA were 93.5% (43/46) and 89.5% (17/19), respectively. The assay specificities were 98.8% (168/170) and 99.4% (169/170) for in-house anti-SARS-CoV-2 IgG and IgA ELISAs, respectively. The agreement kappa values for in-house anti-SARS-CoV-2 IgG and IgA ELISAs were 0.93 and 0.91, respectively and classified as excellent.

## Discussion

We have developed in-house anti-SARS-CoV-2 IgG and IgA ELISAs using SARS-CoV-2 spike RBD as antigen. Among current diagnostic assays, the nucleocapsid protein (N) and the spike protein (S) are mostly used as target antigens in antibody tests for coronavirus infections. However, previous studies showed that these antigens may not be the optimal targets for diagnosis of SARS-CoV-2 infections. For example, the N protein of SARS-CoV-2 has been shown to be unsuitable for the detection of virus-specific antibodies due to very high levels of cross-reactivity between COVID-19 and SARS patient sera [9]. Similarly, the S2 domain of S also exhibits cross-reactivity to S protein of MERS-CoV [10]. Moreover, while the S1 domain is the most specific antigen for the diagnosis of COVID-19, the protein RBD exhibits greater sensitivity for diagnosis among patients with mild infections [11]. Therefore, we tested the RBD domain of the spike protein of SARS-CoV-2 as the target antigen of in-house anti-SARS-CoV2 IgG and IgA ELISAs. From our in-house ELISAs, optimized OD of IgG and IgA cut-off values at higher than 3.6 and 3.2 fold of mean OD negative, respectively. At these cut-offs, the ELISAs displayed high sensitivity and specificity. When compared with other ELISA assays, the positive cut-off values were concordant with the commonly cut-off used, which was set up at 3 standard deviations (SD) above the mean of the negatives [12,13].

Typically, IgA antibodies are primarily responsible for mucosal immunity and appear during early stages of viral infection. The sensitivity for early diagnostics of SARS-CoV-2 infection might be increased when combining IgA antibody testing with RT-PCR testing [14]. Normally, IgG antibodies predominantly present in the convalescent phase and are responsible for long-term immunity against SARS-CoV-2 infection. Recently, the IgG ELISAs against SARS-CoV-2 showed high sensitivity, more than 90%, for samples collected more than 14 days post onset of symptoms (POS) [15,16]. Nevertheless, Seow *et al*. observed that up to 51.6% of SARS-CoV-2 infected patients developed IgG, IgA and IgM synchronously at the early stage of infection (less than 14 days POS) [17]. Altogether, the combination of IgA and IgG ELISA detections might be useful in terms of maximizing the diagnostic sensitivity of the COVID-19 test.

Diagnostic tests with Emergency Use Authorization (EUA) status from the US Food and Drug Administration (FDA) for COVID-19 include nucleic acid testing, RT-PCR, and serological assays such as Euroimmun anti-SARS-CoV-2 IgG ELISA [18,19]. Validation of our in-house ELISAs used RT-PCR and a commercial, Euroimmun ELISA kits that uses S1 domain as target antigen. The manufacturer reported a sensitivity of Euroimmun anti-SARS-CoV-2 IgG and IgA ELISAs of 94.4% and 96.9%, respectively, when testing serum collected > 10 days POS [20]. The data indicate that the in-house anti-SARS-CoV-2 IgG ELISA exhibits “excellent” performance compared to both RT-PCR and Euroimmun ELISA (Kappa value: 0.93–0.94). Similarly, the in-house anti-SARS-CoV-2 IgA ELISA was classified as “excellent” when compared to Euroimmun (Kappa value = 0.91), though was only considered “fair” when compared to RT-PCR (Kappa value: 0.51). Taken together, these in-house ELISAs could not only detect anti-SARS-CoV-2 IgG and IgA antibodies, but could do so at a level of performance comparable to that of RT-PCR and Euroimmun kits.

It should be noted that the small number of sample used in this study is a limitation, and increasing the size of the serum panel used could strengthen the results. Furthermore, it is important to recognize that an analysis of cross-reactivity of this assay with seasonal human coronaviruses (such as HCoV-229E, HCoV-NL63, HCoV-OC43 and HCoV-HKU1) was not assessed. This could be relevant, since one study showed 10% cross-reactivity among sera from healthy blood donors for SARS-CoV-2 S-reactive IgG antibodies [21]. While relatively low, this cross-reaction between IgG antibodies to the seasonal coronavirus and to the pandemic SARS-CoV-2 virus needs to be evaluated to fully understand the value of the assays developed in this study [22].

Despite these limitations, these in-house ELISAs have several advantages that should be highlighted. The assays are easy to perform and low-cost compared to commercial ELISA kits. Furthermore, this platform can be quickly and easily be adapted and adjusted to be specific in the event of future pandemics by changing only target antigens relevant to circulating SARS-CoV-2 strain or subtype. This assay may prove to be a fast and effective diagnostic to deploy early on in a future SARS-CoV-2 outbreak. Similarly, it can also be adapted by simply changing the capture antibodies which are widely available commercially. However, an assessment of this assay cross-reactivity, especially in response to the extent of new SARS-CoV-2 variants and vaccination, would be required. As such, these in-house ELISAs may be an effective alternative for use in various investigations of SARS-CoV-2. They may prove useful in applications ranging from serological surveillance studies, to vaccine and antiviral therapy efficacy trials.

## Conclusion

The in-house anti-SARS-CoV-2 IgG and IgA ELISAs tested in this study may be useful for diagnosing the infection caused by SARS-CoV-2 to control the spread of COVID-19 and may provide insight into the seroprevalence status of COVID-19.
